# An active approach of pressure waveform matching for stress‐based testing of arteries

**DOI:** 10.1111/aor.14064

**Published:** 2021-09-25

**Authors:** Emmanouil Agrafiotis, Markus A. Geith, Mohammad A. Golkani, Vera Hergesell, Gerhard Sommer, Sotirios Spiliopoulos, Gerhard A. Holzapfel

**Affiliations:** ^1^ Institute of Biomechanics Graz University of Technology Graz Austria; ^2^ Institute of Automation and Control Graz University of Technology Graz Austria; ^3^ Department of Cardiac Surgery Medical University of Graz Graz Austria; ^4^ Department of Structural Engineering Norwegian University of Science and Technology (NTNU) Trondheim Norway

**Keywords:** active compliance chamber, arterial physiology, cardiovascular system, feedback control, in vitro testing of arteries, mock circulation loop, variable compliance

## Abstract

**Background:**

Arterial compliance assists the cardiovascular system with three key roles: (i) storing up to 50% of the stroke volume; (ii) ensuring blood flow during diastole; (iii) dampening pressure oscillations through arterial distension. In mock circulation loops (MCLs), arterial compliance was simulated either with membrane, spring, or Windkessel chambers. Although they have been shown to be suitable for cardiac device testing, their passive behavior can limit stress‐based testing of arteries. Here we present an active compliance chamber with a feedback control of variable compliance as part of an MCL designed for biomechanical evaluation of arteries under physiological waveforms.

**Materials and Methods:**

The chamber encloses a piston that changes the volume via a cascaded controller when there is a difference between the real‐time pressure and the physiological reference pressure with the aim to equilibrate both pressures.

**Results:**

The experimental results showed repeatable physiological waveforms of aortic pressure in health (80–120 mm Hg), systemic hypertension (90–153 mm Hg), and heart failure reduced ejection fraction (78–108 mm Hg). Statistical validation (*n* = 20) of the function of the chamber is presented against compared raw data.

**Conclusion:**

We demonstrate that the active compliance chamber can track the actual pressure of the MCL and balance it in real time (every millisecond) with the reference values in order to shape the given pressure waveform. The active compliance chamber is an advanced tool for MCL applications for biomechanical examination of stented arteries and for preclinical evaluation of vascular implants.

## Graphical abstract

The arterial compliance is responsible for the physiological pressure waveform. In mock circulation loops, this effect is incorporated with compliance chambers passively, generating a non‐physiological waveform. Thus, these approaches are not suitable for stress testing of arteries. The active compliance chamber is presented here reproduces a physiological pressure waveform, via a cascaded controller, that benefits stress‐based testing of arteries.

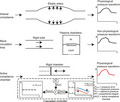

## 1 INTRODUCTION

Arterial compliance describes the dynamic capacity of the vasculature to expand upon pressure alterations and is expressed as a function of vessel volume per change in pressure.^1^ This nonlinear behavior is not adequately emphasized in vitro for stress‐related research of arteries. In vivo, the arterial compliance has three key roles during the cardiac cycle: (i) in the systolic phase it can store up to half‐fraction of the stroke volume by distending the arteries; (ii) the “elastic reservoir” ensures the continuous blood flow during diastole that otherwise is ceasing when the aortic valve is closed; (iii) the distensibility of the arteries regulates the blood pressure forming the physiological pressure waveform.^2^ These factors should be taken into account when simulating the vasculature for biomechanical testing of arteries, for example, aortas with stent‐grafts, or durability testing of stents.

Mock circulation loops (MCLs) are the in vitro representation of the human cardiovascular system for testing medical devices and implants.^3^ In the past, compliance approaches in MCLs have been associated with the evaluation of medical devices such as ventricular assist devices and total artificial hearts.^3^ These are described either with membrane‐based,^4^, ^5^, ^6^ spring‐based,^7^, ^8^ or Windkessel compliance chambers.^9^, ^10^, ^11^, ^12^, ^13^, ^14^, ^15^ Either approach returned MCL performance improvements closer to physiological values, but they do not have complete control over the shaping of the in vivo waveform. While these are suitable compliance tools for evaluating cardiac devices, there are several reasons why these approaches may not benefit from physiological testing of arteries in MCLs.

Particularly, membrane compliance chambers employ elastic membranes that divide the circulation from an air compartment. Hence, the compliance is dependent on the bulging effect of the material and the pneumatic pressure in the sub‐chamber. In a study^5^ that involved a nonlinear material as a membrane, a physiological amplitude was reported, but the shape of the aortic pressure (AoP) waveform is almost linear. This might derive from the membrane's limited behavior: inward or outward protrusion paired with systole or diastole neglecting the interim physiological variations. Another study^6^ employed a silicone membrane as a compliance mechanism to reproduce a physiological AoP waveform, but the chamber could not eliminate the pressure disturbances generated from the mechanical heart valves. Spring‐based^7^, ^8^ chambers substitute the air compartment with a spring. In this case, the response relies on the material properties of the spring, and in higher pressure scenarios where the spring is overstretching, the compliance response is stiffer than naturally. Present oscillations derive from the material behavior of the compliance chamber and from the function of the heart valves that induce disturbances due to opening–closing impulses. These chambers could benefit medical device testing, but issues such as pressure disturbances, inability to shape the pressure waveform, and to simulate complex physiological scenarios call into question their candidacy for stress‐based testing of arteries.

Windkessel chambers are optimal compliance candidates because they use compressible air instead of a membrane/spring. Moreover, they adopt either a three‐element^9^, ^10^, ^11^, ^12^ (venous, arterial compliances, and vascular resistance) or a five‐element^3^, ^14^, ^15^ (venous, arterial compliances, characteristic and vascular resistance, and inertia) Windkessel. They are closed chambers compartmentalized by liquid and air that is compressed/expanded during systole/diastole, respectively. However, Windkessel chambers must have adequate air volume capacity according to the perfect gas law, stating that in an isothermic expansion/reduction the pressure–volume relationship is constant.^16^, ^17^ An advanced approach by Timms et al.^3^ mitigated this limitation using a multi‐Windkessel setup with resulting AoP in agreement with physiological waveforms. In a later study,^14^ an improved setup was achieved by implementing into a large solely Windkessel chamber a piston to regulate the air volume capacity. The authors^14^ injected compressed air, and together with the fluid‐level threshold, this resulted in an enhanced regulation of the AoP. In a recent study,^18^ the AoP has been simulated by optimized Windkessel chambers with predefined air volumes with an option to plug variable compliance units to alter the chamber volume, but the authors did not report its mechanism. A representative published Windkessel approach^19^, ^20^ uses pneumatic proportional solenoid valves to regulate the pressure inside the compliance chamber. Nevertheless, the impulse mechanism of these valves might induce an interfering pressure signal, and the accuracy of the AoP is not clear since the authors do not demonstrate an individual waveform. Amabili et al.^21^ measured arterial diameter‐based compliance of descending aortas in an MCL employing Windkessel chambers under different heart rates (HRs). They observed physiological AoP waveforms and shifts in compliance with increasing HRs. To summarize, Windkessel approaches reproduced the AoP waveform satisfactorily enabling accurate evaluation of medical devices. Yet, due to the predefined Windkessel volume, the ARC regulation remains passive; a matter that could restrict stress‐related testing of arteries that mandate accurate waveforms.

In every cycle that pressure oscillations occur and the pressure waveform deviates from the physiological shape, the resulted waveform is nonphysiological. Nonphysiological waveforms overstress the arterial wall in the circumferential, radial, and longitudinal directions. A nonphysiological stimulus leads to a supra‐ or sub‐physiological shear stress within the arterial tissue layers. Since the arterial tissue layers are highly sensitive to stresses, the triggering of such a stimulus^22^ switches the cellular phenotype and induces remodeling of the extracellular matrix. Matrix transformation affects the mechanical response of the tissue and by bearing the load progressively the vessel is stiffening. This could lead to a deterioration of the arterial wall,^23^ and possibly invalid tests and misleading results. From a biomechanical perspective, stress testing of arteries benefits from an environment where the pressure waveform is physiological, the compliance chamber could eliminate oscillations, and the pressure–volume relationship in the chamber is controllable. Deviations from the physiological waveform might not affect the evaluation of medical devices, yet it could be critical for soft biological tissue and its biomechanical outcomes. Of importance is their minimization when testing blood‐contacting devices such as vascular stent‐grafts on arteries with the purpose to investigate the mechanical response of the tissue.

In order to make a leap from the passive to the active state with controllable and variable compliance, an active compliance chamber is introduced as part of an MCL. The MCL is divided into a simulated left ventricle and the arterial segment with mechanical heart valves. The simulated left ventricle provides systolic and diastolic pulsatility, pressure, and volumetric flows. The active compliance chamber is incorporated in the arterial segment to regulate the pressure waveform of arteries during testing. It regulates the arterial compliance by dynamically shifting the volume of the chamber. This is accomplished through a piston that is tuned, via a cascaded controller, to allow highly controllable responses of compliance. In response to the physiology of the simulated left ventricle, the compliance chamber enables nonlinear variable compliance and can shape the reference pressure in real time. It can be defined as a Windkessel with three elements,^24^ with a single chamber automatically regulating arterial pressure and completely excluding air.

The present study aims to validate an active controller of the active compliance chamber and presents a novel setup for mechanical testing of arteries in vitro. Particularly, here we analyze an integrated controller that can reproduce any given pressure waveform to its reference shape. This can be beneficial when assessing arteries (e.g., thoracic aortas with endovascular stents) and the impact of pressure variations: (i) healthy as a baseline, (ii) hypertension as a major risk factor for vascular diseases,^25^ and (iii) HFrEF to compare the tissue response at a sub‐baseline pressure. For this reason, the aortic pressure was chosen to be reproduced in this study. We also demonstrate the reproduction of the above scenarios and how the active compliance chamber regulates the shape of the waveforms. Τhe proposed chamber could be used to mimic sophisticated and unusual waveforms resulting from aortic valve abnormalities or aortic stenosis. In contrast to conventional MCL approaches, the present setup is intended as a platform for stress‐based tests of arteries and durability tests of stents.

## 2 MATERIALS AND METHODS

### 2.1 Experimental setup

**FIGURE 1:**
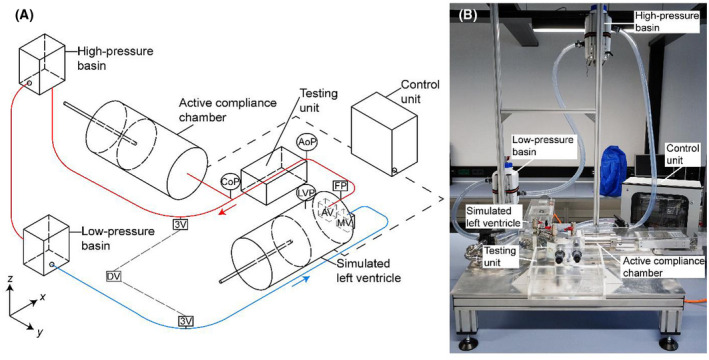
(A) Schematic illustration of the active compliance chamber, which is integrated in the mock circulation loop (MCL). The arterial segment is shown with a red curve and the venous segment with a blue curve. The arrows indicate the direction of flow. 3V, three‐way valve; AoP, aortic pressure; AV, aortic valve; CoP, compliance pressure; DV, drain valve; FP, flow probe; LVP, left ventricular pressure; MV, mitral valve. (B) Photograph showing the complete setup of the MCL

To realize the performance of the compliance chamber, it was necessary to develop an MCL capable of reproducing the cardiac cycle of systole and diastole, the heart rate (HR), and the stroke volume. This was achieved by placing a servo‐driven piston cylinder (AM8022, Beckhoff Automation GmbH, Verl, Germany), namely simulated left ventricle to provide the left ventricular pressure (LVP) and a pulsatile flow into the circulation (Figure 1). Naturally, pressure‐flow variations during systole–diastole in the left ventricle change the amplitude and pressure of the arterial wave. For a realistic ventricular–arterial interaction in vitro, the intermittent flow generated by the simulated left ventricle was directed by two artificial heart valves (500FA27, ATS Medical Inc., Minneapolis, USA), acting as aortic and mitral valve. The simulated left ventricle operated via a multi‐threaded spindle with a pitch of *P* = 3.5 mm, a piston diameter of *D*_piston_ = 50 mm, a cylindrical length *l* = 80 mm, a maximum rotary speed *u*_rot_ = 8000 rpm, and a maximum flow rate *Q*_max_ = 54 L/min. The MCL was capable of reproducing HRs up to 250 beats per minute (bpm).

A transparent testing unit with a heated coil was designed to submerge samples in a liquid bath at 37℃ (e.g., stented aortas). Therein, long‐term testing of perfused arteries examining the biomechanical response of the arterial wall under physiological conditions is feasible. Pressure data were recorded in the simulated left ventricle (LVP), in the arterial segment (AoP), and the compliance chamber (CoP). Pressure sensors (BSP0052, Balluff GmbH, Filderstadt, Germany) with a measuring range of −750 to 1500 mm Hg and an error of ±11 mm Hg (0.5%) were used. A mechanical flow probe (Pro Turbine Flow Meter, RS, Corby, UK) was used ranging up to 30 L/min with an error of ±0.3 L/min (1.0%) after the aortic valve. Attached to the arterial segment, the active compliance chamber was placed to accommodate pressure oscillations during the cardiac cycle and continuation of flow during diastole. The connecting red curve in Figure 1A is denoted as the systemic arterial segment of the circulation. The circulatory flow continued through the high‐pressure basin toward the low‐pressure basin with venous compliance and venous return (blue curve in Figure 1A). The high‐pressure basin is defining the end‐systolic (ESP) and the low‐pressure basin the end‐diastolic pressure (EDP). The basins were adjusted to a specific elevation setting the baseline amplitude for every arterial pressure and the resistance of the system due to gravity. For the AoP, the ESP was elevated at 120 cm and for EDP at 20 cm above the *xy*‐plane. It matched the amplitude for the healthy state and remained the baseline for hypertension and HFrEF scenarios. This made it possible to just change the stroke profile of the simulated left ventricle to reproduce different scenarios without changing the elevation of the basins. The flow travels from the arterial to the venous segment and mimics the peripheral vascular resistance by lowering the systemic pressure according to Bernoulli's law^26^ and the third element of the Windkessel.^24^

All parts were made of E316L stainless steel, and the circulation consisted of polyvinyl chloride tubing with an inner diameter of 25 mm. The working solution was glycerin dH_2_O 50.6% (v/v) (Carl Roth GmbH, Karlsruhe, Germany), a blood analog that matches the viscosity and density of the blood at 37℃.^27^ The solution was poured into the low‐pressure basin until it was filled with 4 liters, acting as a reservoir, providing a circulatory volume of 1.6 L. This basin was equipped with a second heating coil to pre‐heat glycerin (37℃) before the simulated left ventricle began to pulsate and fill the MCL. Air trapped in the circulation escapes in the high‐pressure basin. The pulsatility lasted 10 cycles until the MCL reached equilibrium: filled with liquid and free of air. A simple drainage was achieved by arranging three‐way valves (3V) and a drain valve (DV) in the negative *z*‐direction below the *xy*‐plane (Figure 1A). Figure 1B is a photograph showing the complete setup of the MCL.

### 2.2 Active compliance chamber

The compliance chamber was designed similarly to the simulated left ventricle with a servo‐driven piston (AM8022, Beckhoff Automation GmbH, Verl, Germany) enclosed in a cylindrical chamber, and it was connected directly to the MCL to in order to regulate the pressure of the arterial segment (the distance of the chamber from the arterial segment in Figure 1A is for illustrative clarity). It is filled with glycerin, with the exception of the air compartment in contrast to the Windkessel chambers. It was placed after the testing to maintain partial diastolic flow in the test unit and to aid in fluid inertia from the previous cycle when the aortic valve is closed; this was achieved by controlling the displacement of the piston toward the negative *y*‐direction. Since the *xy*‐plane is a closed‐loop, the pressure in the arterial segment is also evenly distributed. Pressure data at the inlet of the testing unit (AoP) and at the compliance chamber (CoP) have the effect that the function of the compliance chamber influences the arterial segment equally. On this basis, the chamber was connected to the pressure sensor between the simulated left ventricle and the testing unit in order to reform the pressure waveform before the tested subjects and avoid delays. In this way, turbulences between the simulated left ventricle and the testing unit that might affect the aortic valve function or induce wall stresses when simulating extreme scenarios can be avoided. Furthermore, the justification for adding the active compliance chamber in the arterial segment is the need to reproduce the compliance similar to in vivo vessels. This means that the valve function and the ARC must be provided for pressure‐controlled mechano‐responses in both systole and diastole. Furthermore, the use of the active compliance chamber in the arterial segment enables the mimicking of sophisticated and unusual waveforms (e.g., aortic valve complications or aortic stenosis).

The chamber was linked with a high‐resolution (±0.1 μm) position feedback encoder (EKM36, Sick AG, Waldkirch, Germany) that controls the movement of the piston. The piston diameter was *D*_piston_ = 50 mm, the stroke *l* = 62 mm, and a volume capacity of *V*_ACC_ = 0.120 L. In a distensible system such as arteries, approximately half of the stroke volume is stored in the stretched conduit during systole and flows during diastole.^28^ Therefore, the volume of the chamber can store enough liquid to provide diastolic flow. The presented compliance chamber has three roles: (i) it provides the arterial distensibility in the arterial segment during the cardiac cycle (tubing was considered rigid); (ii) liquid storage; (iii) reduce fluid inertia during diastole when the aortic valve is closed.

**FIGURE 2:**
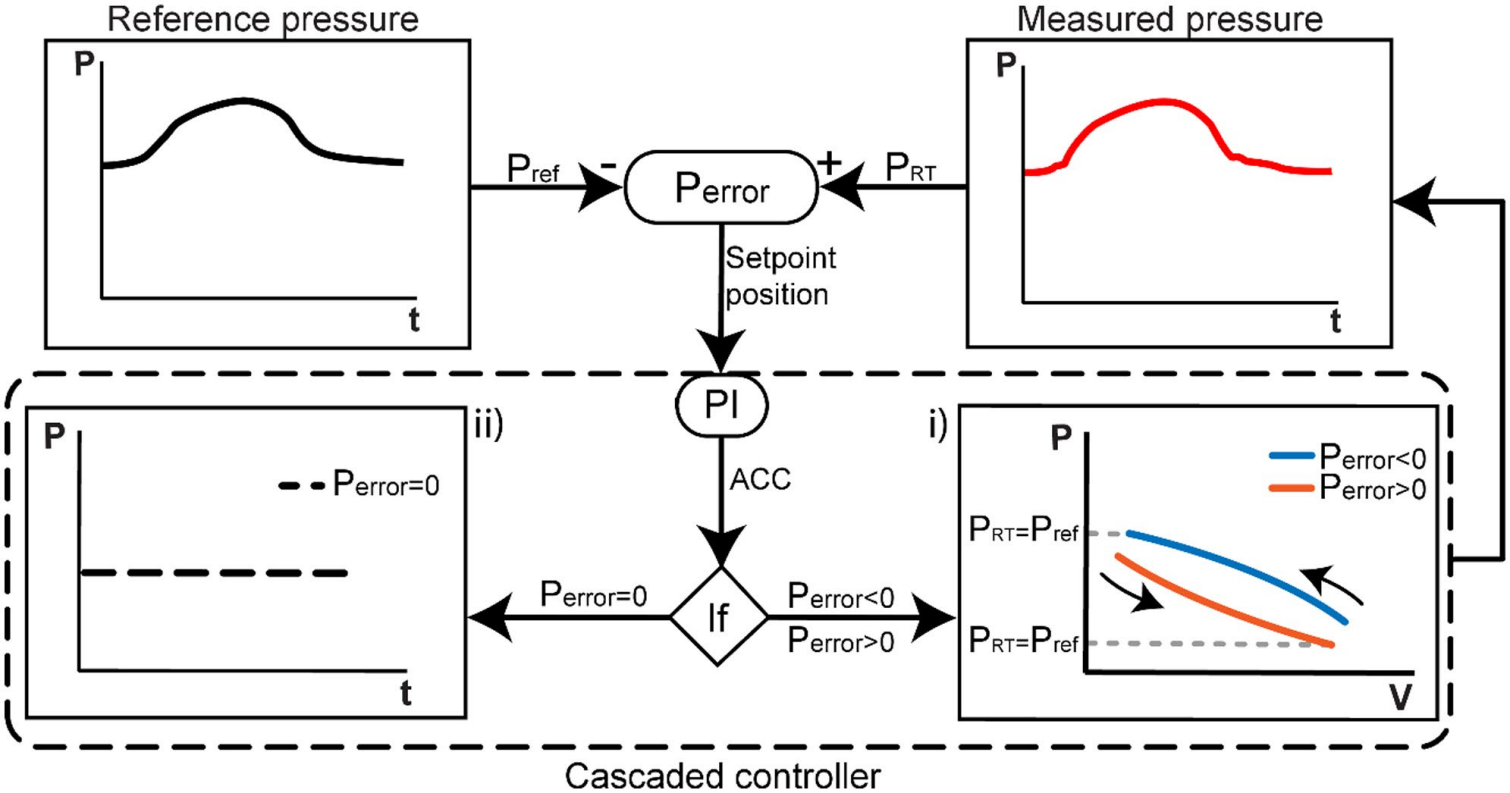
Control diagram of the feedback and cascaded controller. The inputs *p*_ref_ (+ sign) and *p*_RT_ (− sign) lead to *P*_error_ and the control loop sends a signal to the PI and its controllers. Then, depending on the signal, the active compliance chamber (ACC) either (i) expands (*P*_error_ > 0) or reduces (*P*_error_ < 0) its volume until *p*_RT_ = *p*_ref_, or (ii) remains constant (*P*_error_ = 0). The reference pressure *p*_ref_ relates to the target pressure used as input. The measured pressure *p*_RT_ relates to the real‐time pressure of the MCL. The dashed line encloses the functions of the ACC. *P*_error_, pressure deviation; PI, proportional‐integral controller; ref, reference pressure; RT‐pressure, real‐time pressure

The term “active” was so named because of the chamber's ability to automatically regulate the compliance of the arterial segment in accordance with the reference pressure waveform. To control the reproduced pressure waveform in the arterial segment, a feedback control mechanism was implemented (Figure 2). The reference pressure (*p*_ref_) was given as the target pressure. This mechanism builds up a cascaded proportional‐integral (PI) controller (see Control Unit), based on *p*_ref_. The *P*_error_ function checks whether the measured pressure *p*_RT_ differs from the reference pressure *p*_ref_. The measured pressure *p*_RT_ in the arterial segment was used to determine the behavior of the compliance chamber. These values were used as feedback and enable the PI controller to match the reference pressure. If (i) the *p*_RT_ deviates from *p*_ref_, the *P*_error_ is either positive or negative, then the *p*–*V* (pressure–volume) relationship and the compliance of the arterial segment change until *p*_RT_ = *p*_ref_. If (ii) *p*_RT_ = *p*_ref_, the *P*_error_ is zero and the pressure remains constant. Thus, the pressure‐dependent arterial compliance curve is converted into a nonlinear curve and the compliance chamber iteratively reforms the reproduced pressure waveform.

This is possible through the three signals of the chamber‐pressure sensor‐feedback encoder transmitted in the control unit. By definition, the arterial compliance is described as a change in the volume *V* over a change in the pressure *p*, that is C=∂V/∂p.^2^ In this study, the function of the chamber is a combination of the total work done in reversible expansion *W*, by changing the volume *V*,^29^ that is

$$W=‐\int p_{\text{RT}}dV,$$

and the nonlinear pressure‐dependent compliance,^30^ the function is described by

$$‐\frac{\mathrm{dV}}{dp_{\text{RT}}}=C\frac{p_{\text{ref}(t)}}{p_{\text{RT}}},$$

where *C* is the compliance, *dV* is the volume change, and *dp* denotes the pressure change. Due to the rigid walls of the chamber, the vessel distensibility (i.e., stiffness) is characterized by the reversible expansion of the piston and rises a negative slope in the P–V relationship (Figure 2I). The cases of the compliance chamber variability: (i) when *p*_RT_ < *p*_ref_, the *V*_ACC_ is reducing, (ii) when *p*_RT_ > *p*_ref_ the *V*_ACC_ is expanding, and (iii) when *p*_RT_ = *p*_ref_ the *V*_ACC_ is constant (Table 1).

**TABLE 1:** Different cases for the ACC variability

Set value generator	*V*_ACC_	ACC variation
*p*_RT_ < *p*_ref_(*t*)	$\frac{\partial V}{\partial t}<0$	Reduction
*p*_RT_ = *p*_ref_(*t*)	$\frac{\partial V}{\partial t}=0$	Constant
*p*_RT_ > *p*_ref_(*t*)	$\frac{\partial V}{\partial t}>0$	Expansion

Abbreviations: ACC, active compliance chamber; *p*_ref_, reference pressure of the ACC; *p*_RT_, real‐time pressure of the MCL; *V*_ACC_, volume of the active compliance chamber.

### 2.3 Control unit

**FIGURE 3:**
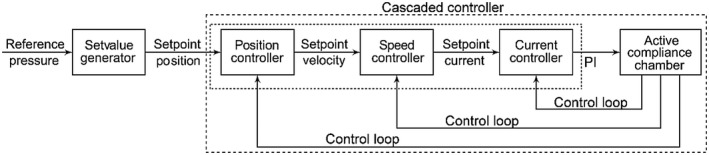
Block diagram of the cascaded control system for the active compliance chamber. The controller creates a movement profile based on the reference input. The position, speed, and current controller consists of a proportional‐integral (PI) controller. Each controller receives signals from the active compliance chamber via the control loop if the actual values deviate from the reference values. The small dashed box indicates the PI controller. The big dashed box indicates the cascaded controller

With a programmable logic controller (PLC) AX5200 (Beckhoff Automation GmbH, Verl, Germany), the operation of both the simulated left ventricle and the compliance chamber was controlled and the measured data were stored. The control concept consists of three cascading controllers (Figure 3) namely the position, the speed, and the current controller starting with the set value generator.

The mechanism builds a movement profile based on the reference function. Each of the above controllers is made up of a PI controller and an anti‐windup scheme. The PI controller can be represented by

$$u\left(t\right)=k_{p}\left(e\left(t\right)+\frac{1}{T_{i}}\int_{0}^{t}e(\tau )d\tau \right),$$

where *u*(*t*) is the output of the controller. The input of the controller denoted by *e*(*t*) is the difference between the measured *p*_RT_ and the setpoint value *p*_ref_, while *k_p_* is the proportional gain, and *T_i_* is the integrator time constant to be tuned. Table 2 summarizes the values that were used.

**TABLE 2:** PI controller constants

Controller	*k_p_*	*T_i_*
Position	1	10
Speed	0.015	0.008
Current	90.3	0.8

Abbreviations: *k_p_*, proportional gain; *T_i_*, integrator time constant.

The anti‐windup scheme is built into the design of the controller in order to mitigate the undesired effect created by an overreaction of the integrator. This effect, which is referred to as “integral windup,” is due to the fact that the large tracking error *e*(*t*) leads to the large signal *u*(*t*). Since this signal cannot be introduced to the system with physical restrictions or safety requirements, the settling time is increased and large overshoots or undershoots or both can occur.^31^ The variable *u*(*t*) of the higher‐level controller is the reference of the lower‐level controller, for example, the position controller produces the speed reference for the speed controller. Chamber's target position, the position that the piston should move to, needs to be introduced to the position controller. The value that indicates the actual position of the compliance chamber is provided by the integrated high‐resolution position encoder.

After a system of units has been used that defines the position zero point and the division of the motor revolutions over a certain number of positioning units, the distance covered can be specified using physical units. If the actual position does not match the position setpoint, the controller outputs a signal that influences the motor so that the actual position reaches the setpoint. This so‐called closed control loop is also used for speed and current control. It should be noted that the actual speed of the piston is also derived from the information of the above‐mentioned encoder. The torque control, with which the torque requirement of the speed controller can be met, is implemented via the current controller. The motor current used for this controller is also measured with high resolution. The cascaded controller leads to motion control, which means that the actual volume in the simulated left ventricle and the actual pressure in the compliance chamber follow the corresponding reference profile.

Within this controllable framework, a PC interface was developed to upload the nonlinear reference functions of pressure *p*_ref_(*t*) and volume *V*_ref_(*t*) of all hemodynamic scenarios (healthy, hypertension, and HFrEF) to PLC and control various hemodynamic parameters (stroke volume, HR, systole, diastole). Similarly, other scenarios can be reproduced. The reference functions include pressure and volume values over time, including systolic–diastolic pressure and stroke volume as presets derived from the literature. In addition, the systole–diastole amplitudes and duration can be varied manually directly at the interface by “stretching” the reference waveforms. Furthermore, the stroke volume is defined as the distance of the simulated left ventricle piston that is to be covered according to the closed control loop described by the position‐based controller. HR is expressed in bpm and is inversely correlated with the duration of the cardiac cycle, that is, when the HR increases, the length of the cycle decreases and vice versa. Scenario transitions were achieved by uploading the respective pressure and volume function during operation. The PLC has automatically transformed the profile *p*_ref_(*t*) and *V*_ref_(*t*). With each transition (e.g., from healthy to hypertension), the MCL changed the movement profile from the simulated LV and the active compliance chamber (Figure 6C–F) to reproduce the target scenario achieved after three cycles while the height of both basins remained the same. The sampling rate for each function was one value per millisecond.

### 2.4 Validation

The compliance chamber offers the possibility to read the actual pressure of the MCL and to correct it with the reference pressure in various hemodynamic profiles. Tested under healthy state, hypertension, and HFrEF, the chamber automatically regulated its position and simulated the specified reference pressure, eliminating valve‐related oscillations. To assess the reliability of the active compliance chamber for long‐term performance, the following experimental procedure was used: the circulation was filled with glycerin until the arterial segment reached a systemic pressure of 75–80 mm Hg. For each profile, the reference volumetric values, HR, and the stroke volume were tuned for the simulated left ventricle, while the reference pressure values were tuned for the compliance chamber. Between the profiles, the compliance chamber required three cardiac cycles for the transition and adapted to the reference pressure. This experiment was repeated four times (*n* = 4) with high steady‐state repeatable results indicating that the compliance chamber achieved the indicated waveforms. In this study, a data cycle for the hemodynamic profiles is graphed to indicate the accuracy of the waveforms. The healthy reference^32^ pressures were digitized using the software WebPlotDigitizer.^33^ The chosen time of the digitized plot was a second or a heartbeat. On the PC interface, a table with 30 time‐points was designed to enter a reference pressure every 0.033 s. Therefore, 30 pressure points were collected from the digitized plot within one heartbeat. The strategy used for the reference waveforms of hypertension and HFrEF was different from that of the healthy waveform because there is no standardized pressure waveform in textbooks. For the hypertension reference^34^ pressures, we extracted the amplitude values (diastolic ≥90 mm Hg – systolic ≥140 mm Hg; HR = 60 bpm) and we “stretched” the curve on the PC interface. Similarly, the HFrEF reference^35^ curve was generated using the amplitude values (78–108 mm Hg).

**FIGURE 4:**
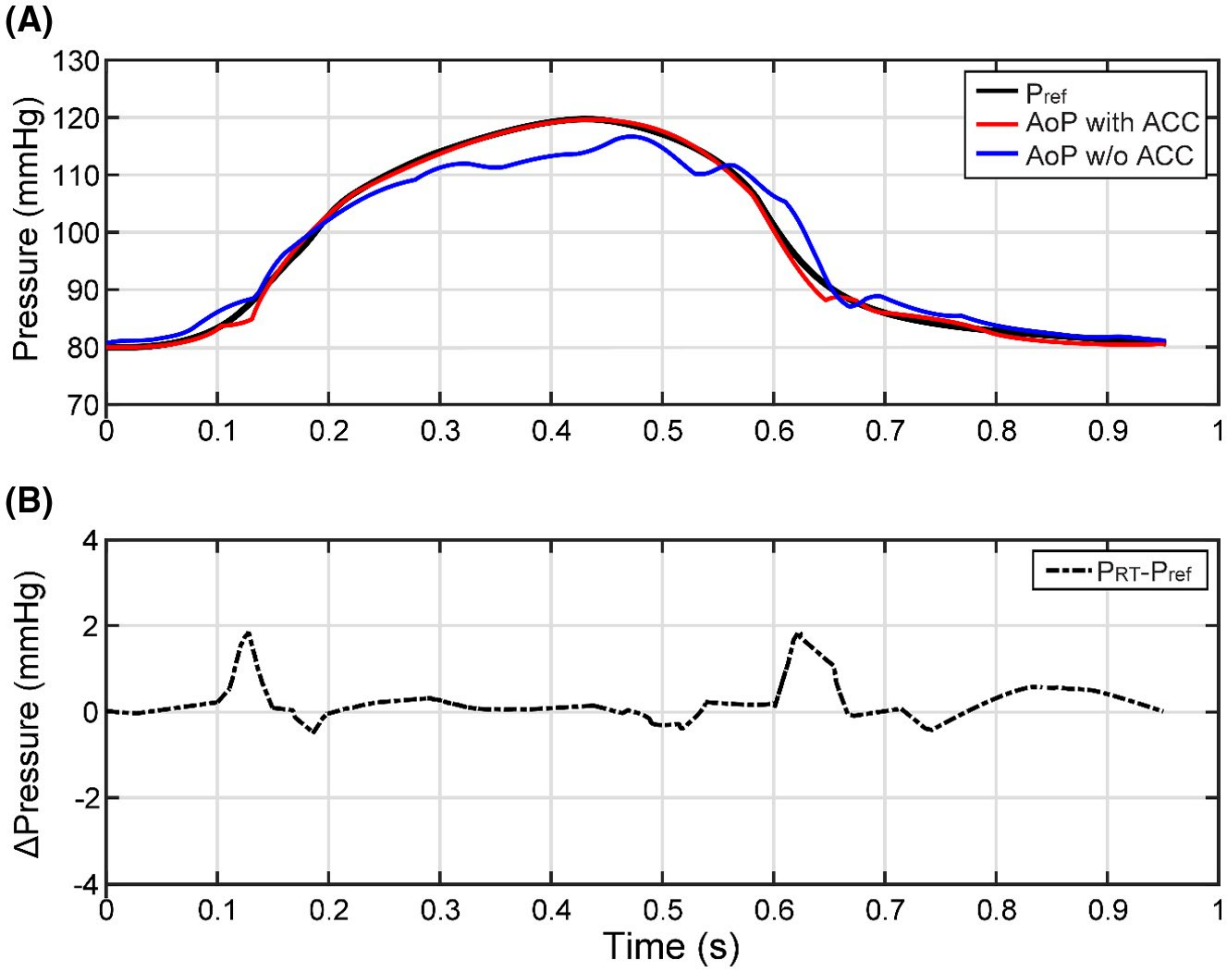
Accuracy of ACC performance: (A) comparison of the *p* reference *p*_ref_ (black waveform), the reproduced AoP with the ACC enabled (red waveform), and the AoP w/o ACC (blue waveform) for the healthy state; (B) tracking error shows ΔPressure of *p*_ref_ – *p*_RT_, which shows the difference between the reference and the measured waveforms. AoP with ACC, aortic pressure with active compliance chamber activated; AoP w/o ACC, aortic pressure with active compliance chamber deactivated; *p*_RT_, measured pressure; *p*_ref_, pressure reference

To validate the accuracy of the active compliance chamber, to track the actual pressure value of the MCL, and to compare it with the pressure reference every millisecond, an evaluation of the tracking error was carried out (Figure 4) The tracking error was calculated in Matlab (Matlab, R2020a, Mathworks, Natick, MA, USA). Further assessment was applied by calculating the mean ($\bar{x}$) ± standard deviation (*σ*) of aortic and compliance pressure of all three profiles (Table 3). Experimental statistical analysis is shown in Figure 4.

**TABLE 3:** Mean $\bar{x}$ ± *σ* of aortic and compliance pressure in all hemodynamic profiles reproduced with the compliance chamber activated

Profile $\bar{x}$ ± *σ*	AoP (mm Hg)	CoP (mm Hg)
Healthy	98 ± 0.3	97 ± 0.5
Hypertension	121 ± 0.7	121 ± 1.4
HFrEF	89 ± 0.8	87 ± 1.2

Abbreviations: AoP, aortic pressure; CoP, compliance pressure.

Compared raw data $\bar{x}$ ± *σ* of the two testing modes, that is, test‐0 (AoP‐ACC deactivated) and test‐1 (AoP‐ACC activated), are plotted to project the significance of the data. For both tests, 20 consecutive cardiac cycles were measured for the healthy state and $\bar{x}$ ± *σ* was calculated. The $\bar{x}$ ± *σ* values of the raw data and the pressure were calculated in GraphPad Prism 8.0 (GraphPad Software Inc., San Diego, CA, USA).

## 3 RESULTS

### 3.1 Validation

Figure 4A shows a comparison graph between the reference pressure^32^ (black waveform) used as an input and the reproduced AoP. The red waveform shows the simulated pressure when the compliance chamber is activated, while the blue waveform shows the healthy state when the compliance chamber is deactivated. This comparison shows the influence of the active compliance chamber on the pressure waveform. By activating the function of the compliance chamber, the oscillatory waveform is converted into the physiological waveform corresponding to the reference. Figure 4B shows the tracking error, the difference between the reference and the reproduced pressure when the compliance chamber is activated, identified as ΔPressure. The tracking error is a validation tool that indicates how precise the function of the compliance controller is in order to balance the actual pressure values *p*_RT_ with the reference pressure values *p*_ref_. It can be seen that the actual and the reference values from the active compliance chamber were balanced throughout the cardiac cycle, except when the valves open/close, which is inevitable.

**FIGURE 5:**
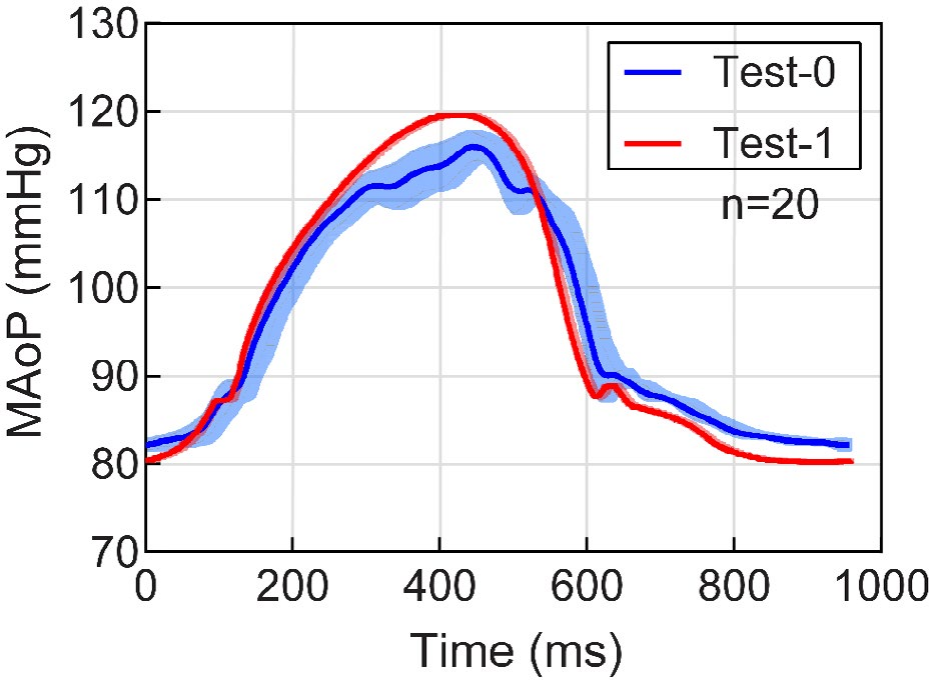
Mock circulation loop repeatable (*n* = 20) healthy raw data with mean aortic pressure (MAoP) $\bar{x}$ ± *σ* versus time, indicatig the accuracy of the ACC controller. Test‐0 is the reproduced waveform (blue) with *σ* (light blue area) and deactivated ACC. Test‐1 is the reproduced waveform (red) with *σ* (light red) and activated ACC. AoP, aortic pressure

The statistical comparison of the measured raw data between the reproduced AoP with activated compliance chamber (red waveform) and with deactivated compliance chamber (blue waveform) is plotted in Figure 5 as the mean AoP. Each cycle (*n* = 20) reproduced a measurement every millisecond. $\bar{x}$ ± *σ* of test‐0 (blue waveform) shows a high *σ* = 2.4 mm Hg (light blue) between cycles. The pressure feedback with the cascaded controller (test‐1) with low *σ* = 0.19 mm Hg (light red) shifted the amplitude and eliminated the oscillatory behavior.

### 3.2 Healthy state

**FIGURE 6:**
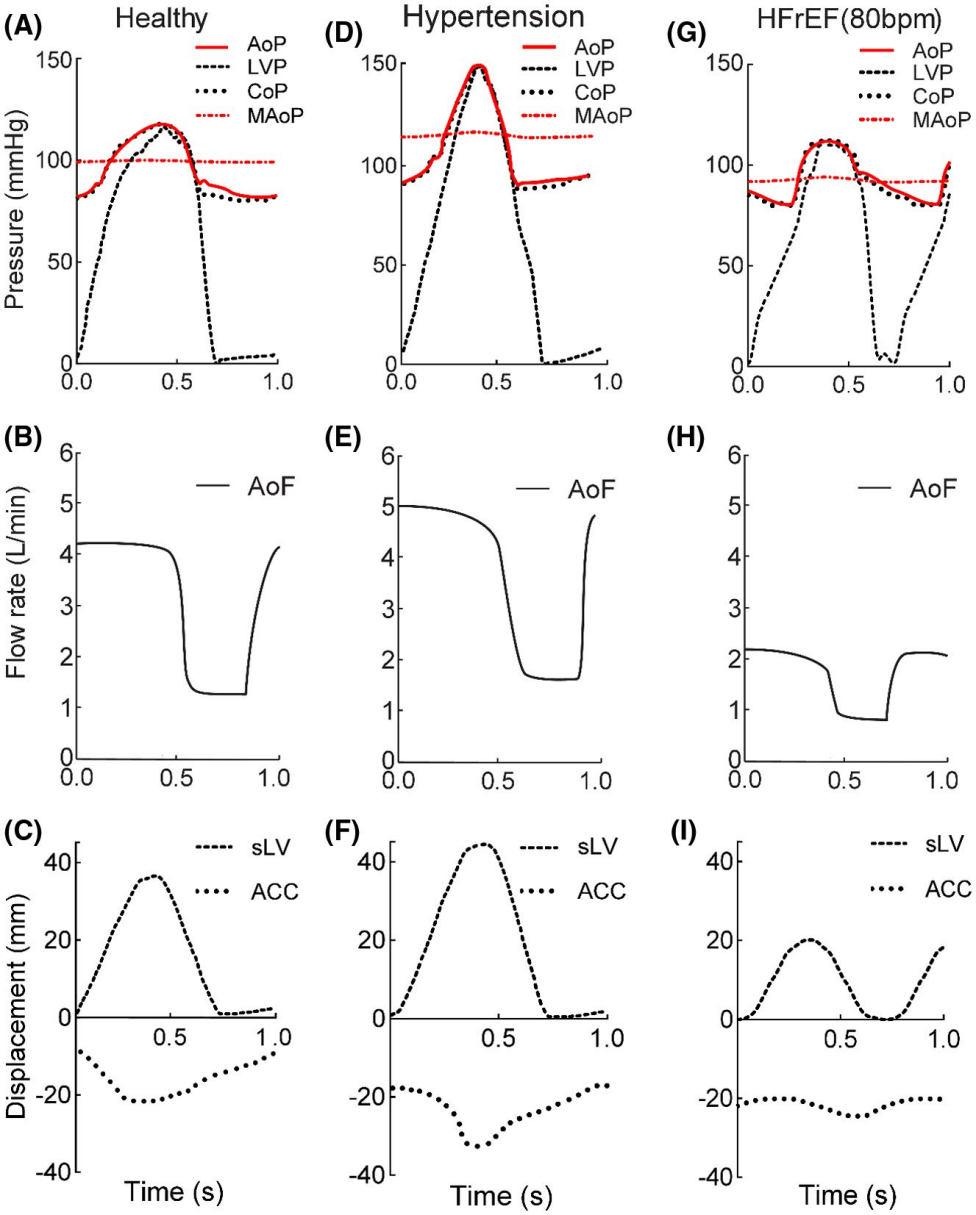
Influence of the ACC on the AoP in the healthy state, (A–C), under hypertension, (D–F), and in HFrEF, (G–I). Measured signals are shown for the pressure‐time, (A), (D), (G), for the flow‐time, (B), (E), (H), and the displacement‐time, (C), (F), (I). ACC, active compliance chamber; AoF, aortic flow; AoP, aortic pressure; CoP, compliance pressure; HFrEF, heart failure reduced ejection fraction; LVP, left ventricular pressure; MAoP, mean aortic pressure; sLV, simulated left ventricle

An optimal physiological condition with an HR of 60 bpm, a stroke volume of 0.07 L/s, and AoP (80–120 mm Hg) was used as a reference.^32^ The reproduced values resulted in a physiological AoP waveform with an ESP of 120 mm Hg, an EDP of 80 mm Hg, and a MAoP of 98.0 ± 0.3 mm Hg (Figure 6A). The AoP and CoP waveforms were the same; a behavior that was observed in the other scenarios. The LVP reproduced an ESP of 120 mm Hg and an EDP of 0.4 mm Hg. Figure 6C shows the displacement profile of the simulated left ventricle to generate the pressure waveforms and the response of the compliance chamber. The compliance chamber adjusts its position to reform the real‐time pressure of the arterial segment to match the reference pressure. The simulated left ventricle displacement defines the amplitude, duration of the systolic–diastolic phase, and the stroke volume amount ejected in each cardiac cycle. For a simulated left ventricle stroke of 36 mm, the stroke volume is 0.070 L, which gives an aortic flow (AoF) 4.2 L/min (Figure 6B) for the 40% of the cycle; the diastolic phase with the aortic valve closed, the compliance chamber stroke was 10 mm in the opposite direction to maintain part of the flow in the arterial segment at 0.020 L/s. This gave an AoF of 1.2 L/min.

### 3.3 Systemic hypertension

The transition to hypertension^34^ (Stage 1: diastolic ≥90 mm Hg – systolic ≥140 mm Hg; HR = 60 bpm) led to an increased AoP of an ESP of 153 mm Hg, EDP of 90 mm Hg, and a MAoP of 121 ± 0.7 mm Hg (Figure 6D). The LVP had an ESP of 151 mm Hg, and an EDP of 1.2 mm Hg. AoF of 5 L/min is achieved with a simulated left ventricle stroke of 42 mm, which corresponds to a stroke volume of 0.082 L for the systolic phase (40% of the cycle) (Figure 6E). The compliance chamber stroke was 16 mm with a partial diastolic AoF of 1.8 L/min.

### 3.4 Heart failure reduced ejection fraction (HFrEF)

Clinically, HFrEF (78–108 mm Hg) has a profile with a lower ESP and a stroke volume ≤50% than of the healthy scenario, followed by an increased HR from 60 to 80 bpm.^35^ The experimental results led to an ESP of 107 mm Hg, an EDP of 77 mm Hg, and a MAoP of 89.0 ± 0.8 mm Hg (Figure 6G). The EDP of the LVP showed 1.4 mm Hg. For the HFrEF, the simulated left ventricle went over to a lower displacement (20 mm), with a stroke volume of 0.036 L giving an AoF of 2.2 L/min (Figure 6H). In this scenario, the behavior of the active compliance chamber was different in the sense that a slight increase in displacement was observed during systole, while the simulated left ventricle stroke was much less to match the HFrEF‐ESP (*p*_ref_ = 107 mm Hg). The compliance chamber stroke moved in the opposite direction compared with the previous two scenarios (Figure 6I). During diastole, the compliance chamber retracted 6 mm to give a partial AoF of 1 L/min.

## 4 DISCUSSION

Conventional MCLs are intended for the evaluation of active and passive cardiovascular implants. The setup presented is inspired by the principles of MCLs with a modified compliance chamber aiming at testing arteries and stents from a biomechanical perspective. This study validates an active approach of a compliance chamber to simulate the arterial compliance in vitro integrated into an MCL. We have presented how the nonlinear compliance of the compliance chamber can reform the arterial pressure in its reference waveform regardless of the ventricular profile that the MCL is generating. The advantage of the active chamber is that the pressure‐volume ratio of the arterial segment is adjusted until the actual pressure is balanced with the reference pressure. Therefore, the volume of the compliance chamber, as it occurs in the Windkessel chambers, does not have to be predefined. Furthermore, no membrane/spring is required as the piston can move freely in the chamber and regulates the volume automatically. Defined as a three‐element Windkessel with nonlinear arterial compliance and adjustment of the resistance of the system to gravity. The three‐element Windkessel approach can adequately embody the arterial system and the pressure–volume relationships compared with its advanced successors, as described in detail by Westerhof et al.^24^ As far as the authors are aware, a compliance chamber with a nonlinear influence on the pressure waveform with the aim of testing stented aortas has not yet been addressed.

Biomechanical examination of arteries, stented or nonstented, requires accurate and controllable pressure waveforms to study arterial changes in the wall that are solely influenced by pressure variations and stenting. Testing arteries in a healthy state (baseline), hypertension, reduced systolic pressure (HFrEF) could provide new insights into how the stented arterial wall is affected and how supra‐ and sub‐physiological stresses can change the matrix homeostasis of the arterial tissue under these pressure shifts. Systemic hypertension was chosen because it is considered to be one of the main risk factors for vascular diseases such as aortic aneurysms treated with endovascular stent‐grafts.^25^ In comparison, an in vitro simulation of the reduced systolic pressure (HFrEF) could provide information on whether there is a remodeling antithesis of the aortic wall. However, the versatility of the active compliance chamber is not limited to these physiological scenarios or to arterial pressure (e.g., AoP). It can simulate the arterial compliance of various arteries and various physiological scenarios that are not shown here for the sake of brevity.

While MCLs with passive compliance approaches rate cardiac devices satisfactorily, they may not be suitable for stress‐based artery testing or stent durability testing. So far Pantalos et al.^8^ reported on one of the first modern approaches to MCLs that involve systemic compliance. They implemented a spring‐based chamber in which the spring constants were set inversely proportional to arterial compliance. They showed peaked physiological aortic pressure waveforms, presumably due to the force response of the springs. Sunagawa et al.^36^, ^37^ published two relevant studies: one deals with simulated arterial compliance^36^ while the other deals with a servo pump system and feedback control^37^ for a ventricular volume servo pump. Similar to the current approach, a linear motor pump with a position encoder with integrated mixed digital and analog feedback control was developed. The authors presented different simulated arterial compliance on the pressure‐flow waveforms of an excised canine ventricle. They implemented a mathematical model of the circulation that was updated at each time step and then used it as feedback to control the volume of the linear pump. The sampling rate of the controller was every two milliseconds, whereas in the present study it is one millisecond. Taylor et al.^5^ embedded a latex membrane in a Windkessel chamber. Despite the nonlinear nature of the membrane, the control accuracy was limited, resulting in a nearly linear pressure waveform. Timms et al.^9^ used a passive arterial Windkessel with a certain volume of trapped air that could reproduce the aortic pressure, but the chamber's inherent compliance could limit the generation of extreme physiological waveforms. In a later report,^3^ the same group optimized their functionality by embodying a five‐element Windkessel with physical loop properties that gave physiological pressure waveforms. Another study^14^ introduced an active Windkessel in which a piston was used to regulate the volume of air in the chamber in incremental steps. This approach resulted in physiological aortic pressure waveforms; small disturbances occurred, which were possibly due to the step‐like piston movement. An advanced system^20^ with controllable pressure responses for testing active and passive cardiovascular devices has recently been described. The implemented chambers control the aortic pressure pneumatically based on the pressure feedback. This enables active adjustments to pressure waveforms and variations in compliance. In another MCL setup with Windkessel chambers, they tested descending aortas^21^ and Dacron aortic grafts^38^, ^39^ in separate studies to examine diameter‐based compliance in different HRs with precise pressure waveforms.

For the intended purpose, the developed compliance chamber is viewed as an advance over the previously mentioned membrane‐based approaches and the typical Windkessel chambers. Because of the complex nonlinear properties of the arterial wall, the P–V relationship can be quite complicated. Translating this relationship in vitro is quite difficult given the rigid walls of the MCL. Instead, the complex viscoelastic properties of the arterial wall that shape the pressure waveform can be simplified. In our setup, the controller actively simulates arterial distensibility by changing the P–V relationship of the arterial segment based on the pressure feedback. This is achieved by moving the piston of the compliance chamber, which is directly connected to the circulatory flow of the arterial segment and whose function forms the arterial waveform. The cascaded controller drives the piston quickly and converts the excess or drop in pressure into a volumetric shift until the reference pressure is reached. Physiological wave reflection in the native arterial circulation contributes both to aortic pressure waveforms and to ventricular‐vascular coupling.^40^ Typical Windkessel models are not able to reproduce this important phenomenon, but the pressure waveform matching approach presented here is able to reproduce pressure waveforms that arise from wave reflection in the real circulation. Another advantage of the current approach is that the feedback control allows the desired pressure waveforms to be simulated, even when wave reflections occur inside the compliance chamber. In other words, regardless of how the pressure is generated in the chamber, the chamber will actively convert the reproduced pressure to the reference pressure.

In contrast to the human vasculature, the negative slope of the P–V relationship in the compliance chamber results from the reversible expansion of the rigid chamber. To better understand the movement profile of the controller, the displacement profiles of each hemodynamic scenario are shown in Figure 6C,F,I. The movement of the simulated left ventricle creates the LVP profile (Figure 6A,D,G) of the MCL. The cascaded controller of the compliance chamber reads the pressure and adjusts its position to establish the reference pressure. It should be noted that the HR, systolic duty, and amplitude of the waveform vary based on presets derived from the literature. The transition in the movement profile from the healthy state to hypertension showed a larger shift in stroke, while the transition to HFrEF expressed a smaller shift. The MCL was found to be highly controllable and repeatable, but only one cycle is graphed to demonstrate the accuracy of the compliance chamber. Precise pressure curves with “smooth” waveforms are in favor of stress‐based artery tests, even if the amplitude is higher or lower than the physiological range (see pressure sensor accuracy ±11 mm Hg). In contrast, long‐term testing under waveforms with spikes and oscillations can compromise arterial integrity. The flow probe (accuracy of ±0.3 L/min) in this study was implemented to get a sense of the flow coming from the ventricle toward the arterial segment and does not affect the results as the focus is on reproducing accurate pressure waveforms for testing of arteries.

Here we analyzed the operation of the MCL in relation to three specific hemodynamic scenarios, namely healthy, hypertension, and HFrEF. In addition, this study validates the performance of the ACC in response to HRs of 60–80 bpm generated by the sLV. However, the full physiological range of the cardiovascular system can reach 200 bpm. In the present configuration, the sLV enables reproduction of up to 250 bpm by inversely correlating the duration of the cycle to accommodate the desired beats, raising a cardiac cycle of 4 × 10^−3^ s. On the other hand, the ACC responds via the cascaded controller, which has a current control cycle of up to 6.2 × 10^−5^ s for dynamic positioning (see Figure 3). Such rapid responses of the controller are sufficient to cover the full physiological range. In order to avoid pressure instabilities inside the chamber, which could arise due to the dynamic shifts, the diameter of the ACC was deliberately designed at *D*_piston_ = 50 mm in order to enable the desired pressure shifts with small positioning increments. Since the blood pressure spectrum is relatively low, the positioning increments shape the physiological waveform appropriately. It should be noted that the ACC performed with similar accuracy up to 160 bpm. In terms of control, the capabilities of the ACC can also cover 250 bpm, but this has not yet been tested.

In order to study the mechanics of arterial tissue, it is necessary to mimic the in vivo vasculature. This means that the arterial segment should provide systole–diastole, aortic valve function, and compliance. In the present MCL, the location of the compliance chamber is controversial. Currently placed after the testing unit, the pressure is evenly distributed in the arterial segment due to the rigid tube, and the arterial compliance can be simulated. This can be seen in Figure 6A,D,G, where pressure data before the test unit (AoP) and after (CoP) show the same waveforms. In contrast, placing the compliance chamber between the simulated LV and the testing unit can lead to turbulence, undesirable interactions with the valvular function and between the coupling of the simulated left ventricle and the compliance chamber. Last but not least, the stability of the controller can be impaired in extreme scenarios. Motivated by this fact, the compliance chamber was placed in the arterial area after the test unit, where it could provide diastolic flow and minimize stagnant areas. In general, the rigid tubes and connections are unavoidable and this limits the regulation of fluid inertia.^24^ The diastolic flow (AoF) was observed in part from the displacement of the ACC piston in the opposite direction and partly from the fluid inertia from the previous cycle, shown in Figure 6B,E,H. The purpose of the system is to test arteries, which is why part of the arterial segment changes from a rigid to a partially elastic behavior. The authors hypothesize that the pressure waveform at the sample site is not attenuated due to the loss of energy from the aorta's native Windkessel and fluid inertia from the previous cycle.^41^ However, the damping is not as significant as the pressure dynamics imposed by the actively controlled chamber.

From a biomechanical point of view, testing under nonphysiological conditions changes the loading scenario on the arterial wall depending on the pressure waveform. Between the pressure amplitude and the irregular pressure waveform (e.g., oscillations), the latter has a much greater influence on the mechanical response of the tissue. It promotes an undesired sequence of events that result not only from the pressure‐load (circumferential stretch) but also from flow disturbances (wall shear stress) that synergistically dysregulate the cross‐talk between endothelial and smooth muscle cells. Changes in phenotype therefore lead to arterial wall ramifications.^22^, ^23^ The MCL in the present study demonstrated an improved simulation of the pressure waveform with pressure‐based compliance that can prevent such an undesired cellular phenotype.

Future studies could include stented aortas from human cadavers attached to the testing unit. This allows us to study the mechanical and structural properties of stented arteries under hemodynamical loading, for example at the proximal landing zone, where the stent interacts with the aortic wall and is known to be a prone area for endoleaks and vascular complications.^42^ A comparative study of human arteries before and after the stent could quantify the damage to the stent in the arterial wall and the compliance mismatch of the stented tissue.^42^ In addition, the compliance profile of the stented and nonstented arteries could be observed by monitoring the pressure dynamics before and after the samples. The pursuit of a quantitative stent‐triggered tissue damage analysis would provide better insights for further improvements in vascular prostheses. By monitoring the hysteresis loop of the pressure versus the behavior of the arterial diameter, the age‐dependent stiffness profiles of the arteries and the pressure‐related compliance changes can be extracted.^43^

The aim of this study is to present an MCL for long‐term tests of stented arteries and stent‐grafts from a biomechanical point of view and to validate the function of the active compliance chamber. The versatility of the design allows for a variety of hemodynamic scenarios to be reproduced and for different arteries to be tested, while the compliance chamber allows for different pressure waveforms to be simulated regardless of shape. Physiological shear and nominal stresses in the arterial wall are said to improve the cellular environment toward in vivo phenotype that prevents vascular remodeling/dysfunction. With controlled variable compliance, the MCL can be used to evaluate mechanical vascular grafts, stent‐grafts, and implants that are complementary to arteries that require a compliance gradient.

## 5 LIMITATIONS

This is primarily a control paper and no arterial tissue is involved. The authors do not intend to imply that this MCL is superior to the above systems, but rather to suggest a different approach that is more beneficial for arterial testing. The limitations of this study include a systolic time‐shift of the reproduced LVP with respect to the mechanical setup. As an in vitro setup, it has a pressure feedback function with a closed control loop for the compliance chamber (purpose of the system) and other autoregulatory mechanisms of the human circulation are missing. In addition, the MCL lacks a Frank–Starling mechanism and a variable resistance. It should be noted that its current state is designed for testing arteries and stents and it cannot assist in the evaluation of cardiac devices and other related applications. Partial diastolic flow is also created, but the artery's innate flow storage during systolic expansion compensates for diastolic flow. The experimental setup presented cannot replace clinical or in vivo tests of arteries and stents, but serves as a preclinical evaluator to optimize stents and to provide insights into the reaction of the arterial tissue after implantation. Future studies should use variable peripheral resistance, ultrasonic flow measurement, and pressure sensors with higher accuracy.

## 6 CONCLUSION

The adaptability of the active compliance chamber in real‐time under different hemodynamic scenarios makes it an optimal compliance candidate for stress‐based artery testing. This is achieved by separating the ventricular and arterial segments using a control profile. The compliance chamber responds to the ventricular hemodynamics and modifies the arterial pressure waveform in order to maintain a certain compliance curve. This function enables the assessment of different stent‐grafts and arteries, whose own compliance profile varies. The presented MCL is a valuable tool for researching arteries and stent‐grafts in the biomechanical field. It can be used to test different stent‐grafts and arteries when realistic physiological conditions, loading scenarios, and a pressure gradient are required.

## CONFLICT OF INTEREST

The authors declare no conflict of interest.

## AUTHOR CONTRIBUTIONS

Emmanouil Agrafiotis, Markus A. Geith, Gerhard Sommer, Sotirios Spiliopoulos, Gerhard A. Holzapfel designed concept. Emmanouil Agrafiotis, Gerhard A. Holzapfel performed methodology. Mohammad A. Golkani was incharge of software. Emmanouil Agrafiotis performed data collection. Emmanouil Agrafiotis performed data analysis. Sotirios Spiliopoulos, Vera Hergesell, Gerhard A. Holzapfel were incharge of funding acquisition. Sotirios Spiliopoulos and Gerhard A. Holzapfel did supervision. Emmanouil Agrafiotis prepared manuscript. Gerhard Sommer, Sotirios Spiliopoulos, and Gerhard A. Holzapfel did critical revision of manuscript.
